# Gaps in Infection Prevention and Control in Public Health Facilities of Sierra Leone after the 2014–2015 Ebola Outbreak

**DOI:** 10.3390/tropicalmed6020089

**Published:** 2021-05-26

**Authors:** James Sylvester Squire, Imurana Conteh, Arpine Abrahamya, Anna Maruta, Ruzanna Grigoryan, Hannock Tweya, Collins Timire, Katrina Hann, Rony Zachariah, Mohamed Alex Vandi

**Affiliations:** 1 Directorate of Health Security and Emergencies, Ministry of Health and Sanitation, P.O. Box 529 Freetown, Sierra Leone; Imurana.conteh@alumni.emory.edu (I.C.); mohamedavandi69@gmail.com (M.A.V.); 2 TB Research and Prevention Center, Yerevan 0014, Armenia; arpine_abrahamyan@yahoo.com (A.A.); ruzanna.grigory@gmail.com (R.G.); 3 World Health Organization, P.O. Box 529 Freetown, Sierra Leone; marutaa@who.int; 4 The International Union Against Tuberculosis and Lung Disease, 75000 Paris, France; hannock.tweya@gmail.com (H.T.); collins.timire@theunion.org (C.T.); 5 The Lighthouse Trust, P.O. Box 106 Lilongwe, Malawi; 6 Sustainable Health Systems, P.O. Box 529 Freetown, Sierra Leone; hann.katrina@gmail.com; 7 UNICEF, UNDP, World Bank, WHO, Special Programme for Research and Training in Tropical Diseases, 20 Avenue Appia, 1211 Geneva, Switzerland; zachariahr@who.int

**Keywords:** SORT IT (Structured Operational Research Training Initiative), operational research, antimicrobial resistance, sustainable development goals, IPCAF

## Abstract

Background: High compliance to infection prevention and control (IPC) is vital to prevent health care-associated infections. In the worst 2014–2015 Ebola-affected district in Sierra Leone (Kenema), we assessed (a) average yearly IPC compliance (2016–2018) using a National IPC assessment tool in the district hospital and peripheral health units (PHUs), and (b) gaps in IPC activities, infrastructure and consumables in 2018. Methods: This was a cross-sectional study using secondary program data. Results: At the district hospital, compliance increased from 69% in 2016 to 73% in 2018 (expected minimal threshold = 70%; desired threshold ≥ 85%). Compliance for screening/isolation facilities and decontamination of medical equipment reached 100% in 2018. The two thematic areas with the lowest compliance were sanitation (44%) and sharps safety (56%). In PHUs (2018), the minimal 70% compliance threshold was not achieved in two (of 10 thematic areas) for Community Health Centers, four for Community Health Posts, and five for Maternal and Child Health Units. The lowest compliance was for screening and isolation facilities (range: 33–53%). Conclusion: This baseline assessment is an eye opener of what is working and what is not, and can be used to galvanize political, financial, and material resources to bridge the existing gaps.

## 1. Introduction

“*No one should get sick while seeking care and no health worker should contract disease while providing care.*” Yet, hundreds of millions of patients and health care workers are affected by avoidable healthcare-associated infections (HCAI) [1]. Such infections are also called “nosocomial infections”, which occur in patients when they receive health care or among health care workers as occupational infections [2,3].

Globally, HCAI is a public health challenge affecting over 1.4 million patients, a large proportion being caused by antibiotic resistant organisms [2,3]. A systematic review assessing the burden of HCAI in low- and middle-income countries (LMICs) reported a pooled prevalence of 15.5 per 100 patients, substantially higher than in high-income countries, where this is about 5 per 100 patients [4]. HCAI result in prolonged hospital stays, long-term disability, increased antimicrobial resistance (AMR), high costs for patients and families, unnecessary deaths of patients and health workers, and significant additional costs to the health system [1].

HCAIs can be avoided by implementing multimodal infection prevention and control (IPC) strategies, such as (i) availing supplies to enable implementation of good IPC practices, (ii) education and training of health care workers and key players, and (iii) monitoring of practices, processes, and outcomes and providing feedback, among others [5,6]. Despite the availability of such low-cost measures, compliance to IPC among health care workers remains low, particularly in LMICs, for different reasons. In India, health care workers (HCWs) were more likely to be compliant if they had more IPC experience, were more knowledgeable about transmission of blood-borne pathogens, and were more committed to workplace safety [7]. In Botswana, emergency nurses identified resource constraints to implementing IPC, such as the lack of the necessary hygiene facilities, inadequate equipment and materials, inadequate staffing, and the lack of sustainable in-service education [8].

Achieving high IPC compliance is of critical importance in Sierra Leone. During 2014–2015, the country was one of the West African countries that experienced the worst Ebola virus disease (EVD) outbreak in history. All 14 health districts in the country were affected with over 13,000 cases and 6000 deaths [9]. Health care workers were disproportionately affected, with 300 EVD cases and 221 deaths [9].

Recognizing the importance of IPC, the Ministry of Health and Sanitation (MOHS) of Sierra Leone established, for the first time (in 2015), a national IPC policy and guidelines for implementing IPC activities in public health facilities. The five thematic areas included hand hygiene, adequate protective wear, sharps safety, sterilization, and waste management [9,10]. The National IPC/Water Sanitation and Hygiene (WASH) assessment tool has been introduced, and a dedicated team regularly visits all health facilities as part of routine supervision and monitoring and collates data related to the specific thematic areas to trigger improvements. This team systematically evaluates all district hospitals and a random sample of peripheral health units (PHUs), once a quarter, using a health facility assessment checklist.

As Sierra Leone is an Ebola outbreak-prone country, it is vital to maintain high IPC vigilance and avoid any apathy towards a “business as usual” mode. Achieving high levels of IPC is also one of the pillars of the national action plan to tackle AMR [10], the logic being “one prevented infection is one antibiotic treatment avoided”. The current COVID-19 pandemic where 14% to 35% of infections reported to the World Health Organization (WHO) are among health care workers is an eye-opener on the justification for high levels of IPC [11].

A PubMed search revealed only one study from neighboring Liberia, which showed a 64% IPC compliance one year after the 2014–2015 Ebola outbreak [12]. There is no study in the region that has assessed IPC compliance over a three-year period. The existing data from quarterly IPC evaluations in Sierra Leone provide an excellent opportunity to get a handle on the trends and status of IPC compliance. We thus decided to assess IPC compliance in one of the worst Ebola affected districts in Sierra Leone—the Kenema district. The district is also endemic for Lassa fever, a viral hemorrhagic disease that demands high compliance to standard IPC practice.

The specific study objectives were to assess a) average yearly compliance for the years 2016–2018 in the Kenema district hospital and PHUs in relation to scores on the National IPC assessment tool, and b) gaps in IPC activities, infrastructure, and consumables in 2018.

## 2. Materials and Methods

### 2.1. Study Design

This was a cross-sectional study using secondary IPC data collected under routine program conditions.

### 2.2. General Setting

Sierra Leone is located at the southwest coast of West Africa and shares borders with Guinea and Liberia. The country is divided into fourteen districts and four provinces, with an estimated population of about 7.7 million people, with over 40% residing in urban areas [13]. The country had a decade (1991–2002) of civil conflict that devastated its health system. The health infrastructure is tiered into tertiary hospitals, district hospitals, and PHUs. The PHUs are delivery points for primary health care in the country and include Community Health Centers (CHCs), Community Health Posts (CHPs), and Maternal and Child Health Posts (MCHPs) [14].

### 2.3. Specific Setting

Kenema District was the study site located in the Eastern Province of Sierra Leone with an estimated population of 665,996 [13]. There are 124 functional public health facilities in the district, including one secondary level hospital serving as the regional referral hospital and 123 PHUs (29 CHCs, 32 CHPs, and 62 MCHPs). Kenema was among the worst-affected districts during the 2014–2015 Ebola outbreak with 503 reported cases of Ebola and 265 deaths (53%). In addition, 71 HCWs were infected, of which 51 (72%) died of the disease [15]. The Kenema district was the district that documented the highest Ebola infection rate among HCWs. This was attributed to poor IPC infrastructure and IPC measures prior to the outbreak.

### 2.4. The National IPC Unit

The 2014–2015 Ebola outbreak accelerated efforts to strengthen health systems in Sierra Leone, including the establishment of a MOHS-led National IPC unit. This unit was mandated to provide leadership and to coordinate and monitor the implementation and strengthening of IPC standards in all health facilities. Prior to the Ebola outbreak, no IPC activities existed in Sierra Leone, nor were there any dedicated staff for IPC at public health facilities. By November 2015, health care personnel had received training, and IPC implementation was rolled out to all public health facilities in the country. This was followed by the appointment of district IPC supervisors and an IPC focal person in each health facility. This team was charged with the responsibility of coordinating and implementing IPC activities.

### 2.5. IPC Checklist for Evaluating Health Facilities

This study used data collected using a standardized national assessment tool known as the IPC/WASH health assessment tool developed by the national IPC unit with technical support from WHO and the US Centers for Disease Prevention and Control (CDC, Atlanta, GA, USA). This tool was developed before the launch of the infection prevention and control framework by WHO in 2018 [16] and was used to assess the actual IPC/WASH practices at health facilities based on the written IPC policies and standards. It was also used for risk assessment, root-cause analysis, and strategic planning to improve IPC standards.

The IPC/WASH checklist (2015 version) is a structured, closed-ended questionnaire with a scoring system on 10 thematic areas or components (Supplementary Materials). The 10 thematic areas include (1) availability of screening and isolation facilities, (2) IPC/WASH organization, (3) hand hygiene, (4) personal protective equipment and supplies, (5) sharps safety, (6) decontamination of medical equipment, (7) decontamination of linen, (8) decontamination of the environment, (9) waste management, and (10) sanitation. Each thematic area has sub-component questions with “yes” or “no” responses coded as 1 or 0, respectively.

The checklist has a total of 68 questions for hospital assessment and 64 questions for PHU assessment. For each thematic area, the total number of “yes” responses are added and divided by the total number of questions for that section. This is multiplied by 100 to obtain the percentage score. An overall score is computed for each thematic area, and based on the percentage scores, compliance is graded as (i) compliant: 85% or above (GREEN); (ii) partial compliance: 70 to 84% (AMBER) and; (iii) minimal compliance: below 70% (RED).

### 2.6. Study Population and Period

The assessments included the Kenema district hospital and nine PHUs (three Community Health Centers, three Community Health Posts, and three Maternal Child Health Posts) selected based on convenience per quarter. The study period was January 2016 to December 2018. Field visit teams consisted of 3 to 4 people from the National IPC Unit (NIPCU) of the MOHS, District IPC Focal person(s), and supervisors supported by WHO, who visited the district on a quarterly basis and collated the data. The field teams were health professionals of different cadres (registered nurses, pharmacists, public health officers, and monitoring and evaluation (M&E) officers) with advanced training in IPC, being certified as IPC master trainers and system managers. The supervision process is such that for each quarter, the selected health facilities are visited by the same team covering at most two facilities per day. At each health facility, the team conducted a detailed assessment of the IPC status, completed the IPC checklist, and also provided on-the-job mentorship. To ensure quality, staff from WHO and US Centers for Disease Control were part of the supervision team.

### 2.7. Data Variables, Data Sources, and Validation

Data variables included facility type, facility name, year (and quarters), and IPC component scores, which were all part of the IPC checklist (the primary data source). This data were entered into a Microsoft Excel^®^ spreadsheet at the National IPC unit by dedicated M&E) officers, which was used for analysis. Data validation was done by the principal investigator who took a random sample of 10% of all IPC checklists (paper-based), and these data were compared with those entered in Microsoft Excel^®^. Where there were errors, further elaborate cross-checking was done. However, data for this study were available for one quarter in 2016 and three quarters in 2017 and 2018.

### 2.8. Data Analysis and Statistics

Data were analyzed using descriptive statistics and results expressed as frequencies and percentages. Average scores by year for each IPC thematic area were computed for the district hospital and PHUs and expressed graphically using color codes. To identify specific gaps in 2018, we listed the sub-components with zero scores in IPC activities, consumables, and infrastructure.

For PHUs, we similarly calculated the proportion of facilities with zero scores for each sub-component. Any sub-component with 50% or more zero scores was considered as a significant gap area.

## 3. Results

### 3.1. Average Yearly Compliance to IPC at the Kenema District Hospital

**Figure 1:**
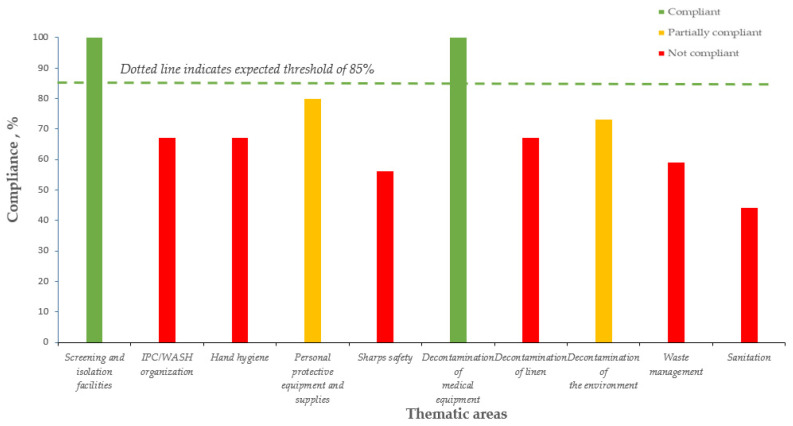
Average yearly IPC/WASH compliance in the 10 thematic areas, Kenema district hospital, Sierra Leone (2018).

Table 1 shows the average IPC/WASH compliance from 2016 to 2018 in the Kenema district hospital. In relation to the overall expected compliance threshold of 85%, the compliance increased from 69% in 2016 to 82% in 2017, and then declined to 73% in 2018. In 2017, six of the ten thematic areas achieved the expected compliance threshold of 85%, while in 2018, only two achieved this threshold. Compliance for screening/isolation facilities and decontamination of medical equipment progressively improved, reaching 100% in 2018. In 2018, the three thematic areas with the lowest compliance were sanitation (44%), sharps safety (56%), and waste management (59%) (Figure 1).

### 3.2. Average Yearly IPC Compliance in Peripheral Health Units (PHUs)

**Figure 2:**
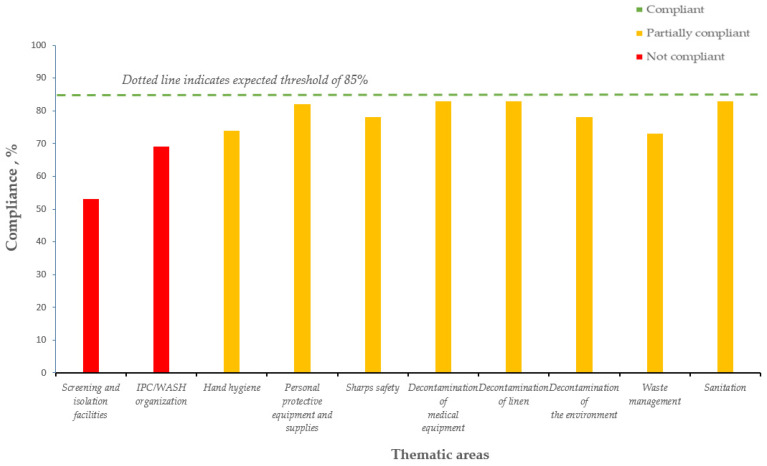
Average yearly IPC/WASH compliance (average of quarterly scores for the year, three quarters assessed) in the 10 thematic areas, Community Health Centers, Kenema district, Sierra Leone (2018).

**Figure 3:**
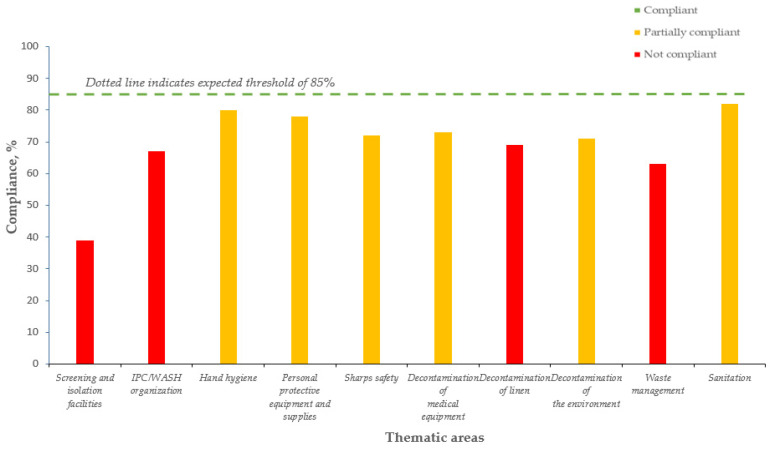
Average yearly IPC/WASH compliance (average of quarterly scores for the year, three quarters assessed) in the 10 thematic areas, Community Health Posts, Kenema district, Sierra Leone (2018).

**Figure 4:**
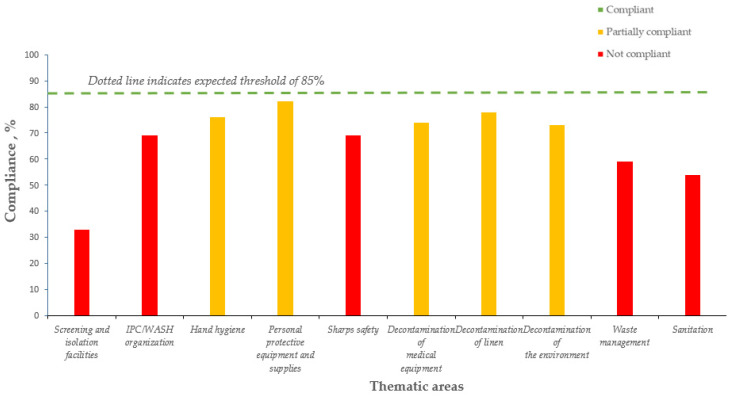
Average yearly IPC/WASH compliance (average of quarterly scores for the year, three quarters assessed) in the 10 thematic areas, Maternal and Child Health Posts, Kenema district, Sierra Leone (2018).

The Tables show the average IPC/WASH compliance from 2016 to 2018 in CHCs (Table 2, Figure 2), CHPs (Table 3, Figure 3), and MCHPs (Table 4, Figure 4).

In 2018, the minimal compliance threshold of 70% was not achieved in two thematic areas for CHC, four for CHP, and five for MCHP. For CHCs, these were screening and isolation facilities (53%) and IPC/WASH organization (69%); for CHPs, this included screening and isolation facilities (39%), waste management (63%), IPC/WASH (67%), and decontamination of linen (69%); and for MCHPs, these were screening and isolation facilities (33%), sanitation (54%), waste management (59%), and IPC/WASH and sharps safety (69% each).

In 2018, the lowest compliance in all three types of PHUs was for screening and isolation facilities.

### 3.3. Gaps in Specific Activities, Infrastructure, and Consumables for Kenema District Hospital and PHUs in 2018

All facilities had gaps in four activities, namely action plan not implemented, waste not segregated at the point of care, waste not disposed of according to appropriate color coding, and no signed record of cleaning available (Table 5).

For infrastructure and consumables, all facilities had gaps in three areas, including no designated laundry area, mattresses without intact waterproof covers or used with a separate mackintosh, and no appropriate bin liners in each bin (Table 6).

At the PHU level, out of a total of 64 questions used to identify gaps in activities, infrastructure and consumables, the maximum number of gaps were in MCHP (39) and CHC (41).

## 4. Discussion

This first study from Sierra Leone shows that following the introduction of a national IPC policy and implementation guidelines, average IPC compliance in health care facilities assessed in Kenema district were generally higher in 2018 compared to 2016. In 2018, the Kenema district hospital and CHCs achieved the minimal compliance of ≥70%, while this was not the case with CHPs (68%) and MCHPs (65%). In 2018, the thematic area with the lowest compliance at district hospital was sanitation, and for PHUs, this was for screening and isolation facilities. Several gaps were identified in activities, infrastructure, and consumables that will need to be bridged.

The study findings are of public health importance as they are an eye-opener to what needs to be done to increase compliance levels and achieve the high expected IPC standards set by the MOHS for health facilities in Sierra Leone. With a new Ebola outbreak lurking around the corner and the ongoing COVID-19 pandemic, the imperative is “today, and not tomorrow”. Considering the added synergy between IPC and AMR prevention, in a manner of speaking, we have an opportunity to hit three birds (Ebola, COVID-19, and AMR) with one stone. Health workers, patients, and the community at large stand to benefit.

The study strengths are that all assessments were conducted by the same dedicated IPC team from the central unit; a standardized checklist was used to assess the various thematic areas, data were cross-validated for quality control, and the subject matter addressed an identified national operational research priority. We also adhered to STROBE guidelines for the conduct and reporting of observational studies in epidemiology [17].

A study limitation is that since the release of the IPCAF tool by WHO in 2018 [18], Sierra Leone also included IPCAF for hospital assessments nationwide. Although our study does not include data for 2019 and 2020, it still provides a useful baseline for further comparisons. Further, due to resource constraints, the assessment of PHUs was done in a convenient sample of only 9 of 129 such facilities, which is a snapshot that may not be fully representative. Resources allowing, future assessments should endeavor to include all health facilities. Another limitation relates to the tool used for assessment, which makes it challenging to compare with other studies and future studies in Sierra Leone using the IPCAF tool, as the overall scores and the grading are different.

Despite these limitations, there are a number of important policy and practice implications. First, while the WHO (IPCAF) [19] tool has a threshold of ≥76% for an advanced IPC level, the MOHS in Sierra Leone set their standards much higher, at ≥85%. This may thus negate the overall grading in Sierra Leone when compared to WHO standards. Our compliance levels must thus be considered with some degree of caution. That said, the higher standards in Sierra Leone were justified as the country is endemic for Ebola, viral hemorrhagic fever, and now COVID-19. Thus, preventing HCAIs, especially those among health workers is crucial [20].

Second, in 2017 (post-Ebola), the Kenema district hospital achieved the expected IPC compliance threshold of over 85% in six of ten thematic areas. This then dropped to two areas in 2018, which might indicate some degree of complacency. However, the hospital still maintained the minimum overall compliance level of >70%, which is commendable. The MOHS-IPC reports for 2019 and 2020 show maintenance of the same level of compliance [21]. In 2018, the two thematic areas that were 100% complaint were screening/isolation facilities and decontamination of medical equipment. This is reassuring for managing hemorrhagic fevers and in view of the ongoing COVID-19 pandemic. In contrast, screening and isolation facilities had the lowest IPC compliance in all the PHUs. This area is thus flagged as needing audit and priority attention. This may be explained by the fact that post Ebola, in early 2016, both temporary screening infrastructure and community volunteers who used to manage these screening activities were no longer available as funding and partner support dried up. Thereafter, most PHUs could not sustain this activity.

Third, there are two weak pillars identified across all facilities and upon which all IPC activities overarch. These include (a) the lack of implementation of the IPC action plans, and (b) IPC/WASH organization. The strength of these pillars is dependent upon supervision, availability of adequate infrastructure and consumables, and understandably, and the inter-dependence between them. For example, without clean water or toilet facilities, the thematic area of sanitation suffers. Similarly, sharps safety would be compromised when close supervision and/or sharps containers are in short supply.

In a resource limited country like Sierra Leone, ensuring that resources needed to assure adequate supervision, infrastructure, and consumables for the hundreds of health facilities in Kenema (and beyond) is a major undertaking that needs to be bridged. One will need to consider both immediate and medium-term measures to tackle the identified problems.

*Immediate measures* needing urgent attention to varying levels between health facilities include the following: ensuring that soap and alcohol hand rub solutions are available at all hand hygiene stations and particularly in clinical areas; ensuring that toilets are functional and provided with a constant source of water; waste triage at source; and adequate waste disposal and sharps disposal. Sierra Leone is currently producing liquid soap and alcohol-based hand rub according to WHO standards using local materials. With the local production of these essential items, we believe the country can afford to ensure all health facilities have liquid soap and/or alcohol-based hand rub solutions. The finding that mattresses in all health facilities did not have water-proof covers needs to be corrected, as such mattresses are impossible to disinfect completely and may become a vehicle for disease transmission.

In the *medium term*, there is a clear need to elaborately review the infrastructure gaps and consumable needs in Kenema district and galvanize support and funding to bridge these gaps. This study provides a good canvas for that work and would also serve for the scale up of such work country-wide. As a start, the top 10 priority gaps in infrastructure and consumables could be decided upon and tackled.

In conclusion, this study provides a good baseline review of the IPC/WASH status in Kenema district and is an eye opener of what is working and what is not. We have identified several gaps in infrastructure and consumables, upon which effective IPC organization and implementation depend. This information will be presented to the MOHS and the One Health AMR committee, including donors in Sierra Leone with a view towards galvanizing political, financial, and material resources to bridge the gaps and act.

## Figures and Tables

**Table 1:** Average yearly compliance in relation to scores on a National IPC/WASH ^1^ assessment tool, in Kenema district hospital, Sierra Leone (2016–2018).

		2016		2017		2018	
	Maximum Score	Score ^2^	(%)	Score ^2^	(%)	Score ^2^	(%)
**Cumulative Score (%)**	68	47	(69)	56	(82)	49	(73)
**Thematic areas**							
Screening and isolation facilities	8	6	(75)	7	(88)	8	(100)
IPC/WASH ^1^ organization	8	8	(100)	7	(92)	5	(67)
Hand hygiene	6	5	(83)	5	(83)	4	(67)
Personal protective equipment and supplies	5	1	(20)	5	(93)	4	(80)
Sharps safety	6	4	(67)	6	(94)	3	(56)
Decontamination of medical equipment	9	7	(78)	8	(89)	9	(100)
Decontamination of linen	6	5	(83)	4	(82)	4	(67)
Decontamination of the environment	5	1	(20)	4	(87)	4	(73)
Waste management	9	7	(78)	5	(59)	5	(59)
Sanitation	6	3	(50)	4	(67)	3	(44)

^1^ Infection Prevention and Control/Water Sanitation and Hygiene. ^2^ Average of quarterly scores for the year (one quarter assessed in 2016; three quarters assessed in 2017 and 2018).

**Table 2:** Average yearly compliance in relation to scores on a National IPC/WASH ^1^ assessment tool in Community Health Centers, Kenema district, Sierra Leone (2016–2018).

	CHC ^2^						
		2016		2017		2018	
	Maximum Score ^3^	Score ^4^	(%)	Score ^4^	(%)	Score ^4^	(%)
**Cumulative Score (%)**	192	143	(75)	388	(67)	433	(75)
**Thematic areas**							
Screening and isolation facilities	24	18	(75)	48	(67)	38	(53)
IPC/WASH ^1^ organization	12	6	(50)	23	(64)	25	(69)
Hand hygiene	18	15	(83)	41	(76)	40	(74)
Personal protective equipment and supplies	15	15	(100)	36	(80)	37	(82)
Sharps safety	18	14	(78)	46	(85)	42	(78)
Decontamination of medical equipment	27	26	(96)	66	(82)	67	(83)
Decontamination of linen	18	11	(61)	33	(61)	45	(83)
Decontamination of the environment	15	11	(73)	23	(51)	35	(78)
Waste management	27	16	(59)	48	(59)	59	(73)
Sanitation	18	11	(61)	24	(44)	45	(83)

^1^ Infection Prevention and Control/Water Sanitation and Hygiene; ^2^ Community Health Center; ^3^ Community Health Centers have a maximum score of 64 per facility. As three facilities were included in the assessment, the maximum cumulative score was 192. ^4^ Average of quarterly scores for the year (one quarter assessed in 2016; three quarters assessed in 2017 and 2018).

**Table 3:** Average yearly compliance in relation to scores on a National IPC/WASH ^1^ assessment tool in Community Health Posts, Kenema district, Sierra Leone (2016–2018).

	CHP ^2^						
		2016		2017		2018	
	Maximum Score ^3^	Score ^4^	(%)	Score ^4^	(%)	Score ^4^	(%)
**Cumulative Score (%)**	192	97	(51)	359	(62)	392	(68)
**Thematic areas**							
Screening and isolation facilities	24	3	(13)	33	(46)	28	(39)
IPC/WASH ^1^ organization	12	6	(50)	19	(53)	24	(67)
Hand hygiene	18	9	(50)	42	(78)	43	(80)
Personal protective equipment and supplies	15	13	(87)	38	(84)	35	(78)
Sharps safety	18	13	(72)	40	(74)	39	(72)
Decontamination of medical equipment	27	23	(85)	56	(69)	59	(73)
Decontamination of linen	18	8	(44)	34	(63)	37	(69)
Decontamination of the environment	15	7	(47)	28	(62)	32	(71)
Waste management	27	12	(44)	45	(56)	51	(63)
Sanitation	18	3	(17)	24	(44)	44	(82)

^1^ Infection Prevention and Control/Water Sanitation and Hygiene; ^2^ Community Health Post; ^3^ Community Health Posts have a maximum score of 64 per facility. As three facilities were included in the assessment, the maximum cumulative score was 192. ^4^ Average of quarterly scores for the year (one quarter assessed in 2016; three quarters assessed in 2017 and 2018).

**Table 4:** Average yearly compliance in relation to scores on a National IPC/WASH ^1^ assessment tool in Maternal and Child Health Posts, Kenema district, Sierra Leone (2016–2018).

	MCHP ^2^						
		2016		2017		2018	
	Maximum Score ^3^	Score ^4^	(%)	Score ^4^	(%)	Score ^4^	(%)
**Cumulative Score (%)**	192	109	(57)	363	(63)	376	(65)
**Thematic areas**							
Screening and isolation facilities	24	3	(13)	40	(56)	24	(33)
IPC/WASH ^1^ organization	12	4	(33)	23	(64)	25	(69)
Hand hygiene	18	12	(67)	41	(76)	41	(76)
Personal protective equipment and supplies	15	15	(100)	40	(89)	37	(82)
Sharps safety	18	13	(72)	41	(76)	37	(69)
Decontamination of medical equipment	27	21	(78)	58	(72)	60	(74)
Decontamination of linen	18	10	(56)	39	(72)	42	(78)
Decontamination of the environment	15	8	(53)	23	(51)	33	(73)
Waste management	27	16	(59)	42	(52)	48	(59)
Sanitation	18	7	(39)	16	(30)	29	(54)

^1^ Infection Prevention and Control/Water Sanitation and Hygiene; ^2^ Maternal and Child Health Post; ^3^ Maternal and Child Health Posts have a maximum score of 64 per facility; as three facilities were included in the assessment, the maximum cumulative score was 192. ^4^ Average of quarterly scores for the year (one quarter assessed in 2016; three quarters assessed in 2017 and 2018).

**Table 5:** Gaps in specific activities for Kenema district hospital and Peripheral Health Units, Sierra Leone (2018). (Blank spaces = no identified gap; × = gap identified).

Activity	District Hospital	CHC	CHP	MCHP
An IPC action plan not developed based on previous assessment feedback			×	×
Action plan has not been implemented	×	×	×	×
Hand hygiene observational audit not conducted in the past 3 months		×	×	×
Report, request, and issue voucher (RRIV) not completed and archived chronologically		×	×	×
PPE supplies are not stored off the floor and in a dry place		×	×	
No sharps injury report for PEP management		×	×	×
Syringes are not discarded in sharps box after single use	×	×		
Sharps containers are filled above the fill mark	×	×		
No policy on cleaning, disinfection, and/or sterilization of medical devices		×	×	×
No delivery set per procedure for the number of deliveries each day		×	×	×
No SOP for handling linen		×	×	×
Clean linen stored on the floor, chairs, or counter tops		×	×	×
Environment not visibly clean		×		
Waste management policy/SOP was not available		×	×	×
Waste was not segregated at the point of care	×	×	×	×
Staff does not handle dirty instruments with rubber gloves, plastic aprons, masks, and gowns			×	×
Waste not disposed of according to appropriate color coding	×	×	×	×
Overfull waste bins in the wards		×		
Clean linen stored on the floor, chairs, or counter tops			×	×
No signed record of cleaning available	×	×	×	×
Stock ordering form not in use at the facility			×	
IPC/WASH committee does not meet every month	×			
Screening form for in-patients in the wards not completed for the last month prior to assessment	×			

CHC = Community Health Center; CHP = Community Health Post; MCHP = Maternal and Child Health Post; PEP = post exposure prophylaxis, SOP = standard operating procedure; PPE = personal protective equipment; IPC = infection prevention and control.

**Table 6:** Gaps in infrastructure and consumables for Kenema district hospital and Peripheral Health Units, Sierra Leone (2018). (Blank spaces = no identified gap; × = gap identified).

Infrastructures	District Hospital	CHC	CHP	MCHP
Facility entrance has no screening stations		×	×	×
Screening station has no single entrance		×	×	×
No designated isolation area		×	×	×
Isolation area not located in a permanent structure		×	×	×
No functional system for grey water drainage and soak away pit		×	×	×
No designated latrines for people with reduced mobility		×	×	×
No hand hygiene station in all clinical areas		×		
No hand washing station in decontamination area		×	×	×
No functional autoclave in use to sterilize critical devices		×	×	×
No designated area/room for cleaning and disinfection of medical devices at the decontamination area		×	×	×
No separate designated area for inspection, assembly, and packaging of medical devices at the decontamination area		×	×	×
Sterile packs not stored in a well-ventilated room/cabinet		×	×	×
No designated laundry area	×	×	×	×
No safe burning pit at the facility		×		×
No functional placenta pit		×		×
Latrines not functional and clean with no constant source of water	×	×		×
No separate toilets for women and men				×
**Consumables**				
No register/form with list of screened individuals		×		×
No hand hygiene posters at all the stations		×	×	×
Interruption of water supply for 1 day or more in the last 1 month		×		×
Stock ordering form not in use at the facility		×		×
Sharp posters were not displayed where sharps are used/prepared		×	×	×
Mattresses has no intact waterproof covers or used with a separate mackintosh	×	×	×	×
No appropriate bin liner in each bin	×	×	×	×
Waste segregation posters were not displayed above all waste bins		×	×	×
Soap or alcohol hand rub not available at all hand hygiene stations	×			

CHC = Community Health Center; CHP = Community Health Post; MCHP = Maternal and Child Health Post; PEP = post exposure prophylaxis, SOP = standard operating procedure; PPE = personal protective equipment; IPC = infection prevention and control.

## Data Availability

The data presented in this study are available on request from the corresponding author.

## Supplementary Materials

The following are available online at https://www.mdpi.com/article/10.3390/tropicalmed6020089/s1, Table S1: National MOHS IPC/WASH standard health facility assessment checklist for PHUs, Table S2: National MOHS IPC/WASH standard health facility assessment checklist for Hospitals.
