# Supplementary material for: Gaps in Infection Prevention and Control in Public Health Facilities of Sierra Leone after the 2014–2015 Ebola Outbreak
Source: Trop Med Infect Dis. 2021 May 26;6(2):89. doi: 10.3390/tropicalmed6020089 (PMC8163185; doi:10.3390/tropicalmed6020089)
Supplement: Supplementary file 1 [file tropicalmed-06-00089-s001.zip › tropicalmed-1179724-supplementary.pdf]

ANNEXES

Table S1: National MOHS IPC/WASH standard health facility assessment checklist for PHUs

GOVERNMENT OF SIERRA LEONE
MINISTRY OF HEALTH AND SANITATION
NATIONAL IPC/WASH PHU ASSESSMENT CHECKLIST

Date of Assessment ____/___/_______ **District** ______ **Chiefdom** ______

Facility Name ______ **City/Town/Village** ______

Name & Designation of Person conducting Assessment ______ **Contact Number** ______ **E-mail Address** ______

Type of facility:

CHC **CHP** **MCHP**

Type of ownership

☐ Government ☐ Private ☐ Faith Based ☐ NGOs

Name of Facility In-charge/Delegate ______ **Contact Number** ______ **E-mail Address** ______

I. SCREENING AND ISOLATION

Instructions:

There should be a screening structure at the entrance to the facility or very close to it – if it is not immediately beside the entrance then score No (0)

Patients should only be able to access the facility by one entrance near or through the screening station – if there is more than one entrance and patients can enter without being screened then score No (0). There should be a hand hygiene station with either soap and water or hand sanitizer at all entrances – if either no soap or no water score as No (0)

	**Standards**	**Score**<br>**Yes=1**<br>**No=0**	**Comments**		
1	There is at least one screening station at the facility				
2.	All facility entrances have a screening station				
3.	The screening station has a single entrance				
4.	There is a hand hygiene station at all facility entry point				
5.	There is soap and water/Alcohol Based Hand Rub (ABHR) at the hand hygiene station				
6.	There is register/form with list of screened individuals				
7.	There is a designated isolation area				
8.	The isolation area is located in a permanent structure				
	**SCORE FOR Qs 1-8:** Total score out of 8	/8	7-8	5-6	<4
	**RAG STATUS**				

II. IPC/WASH ORGANIZATION

Instructions: *IPC and/or Wash focal person - either appointed to hospital or for PHU they know who the IPC focal person for their PHU cluster is or the IPC focal person at the DHMT – if they do not know who the IPC focal person is at the PHU score No (0).*

IPC committees – for Hospitals there should be a hospital committee, for PHUs there should be an IPC committee at the DHMT and the in-charge should be aware of this – if not score No (0).

An internal assessment tool e.g the ward assessment tool is in use as a regular monitoring tool for IPC compliance at the wards.

IPC committee minutes should be available from the Hospital IPC focal person or the DHMT focal person

<table>
<tr><th>#</th><th>Standards</th><th>Score
No=0
Yes=1</th><th colspan="3">Comment</th></tr>
<tr><td>9.</td><td>There is a designated IPC and/or WASH focal person appointed for the facility
- IPC Focal Person □
- WASH Focal Person □</td><td></td><td colspan="3"></td></tr>
<tr><td>10.</td><td>The IPC and/or WASH focal person received training based on the national IPC/WASH guidelines
- IPC only □
- WASH only □
- IPC &WASH □</td><td></td><td colspan="3"></td></tr>
<tr><td>11</td><td>An IPC action plan was developed based on previous assessment feedback and was approved by facility leadership</td><td></td><td colspan="3"></td></tr>
<tr><td>12</td><td>The action plan has been implemented (See report for activity progress)</td><td></td><td colspan="3"></td></tr>
<tr><td colspan="2">TOTAL SCORE for Qs 9-12</td><td>/4</td><td>4</td><td>3</td><td><3</td></tr>
<tr><td colspan="2">RAG STATUS</td><td></td><td></td><td></td><td></td></tr>
</table>

III. HAND HYGIENE

Instruction: *If Hand Hygiene stations are not available in all clinic area, ward areas, and outside single rooms (e.g. labour rooms) -- then score No (0); If only available in some areas then score No (0). Minimum standard is one HH station per 10 patient beds.*

Monitoring Hand Hygiene compliance should be a formal system where opportunities for hand hygiene based on the five moments are observed for a period of at least 30 minutes on a regular basis in all the wards. If it is ad hoc, then score No (0).

A reliable water supply is > three days' supply with system of top up if provided in tanks or a backup system if there is interruption to the main supply.

**#**	**Standards**	**Score No=0 Yes=1**	**Comment**		
13	There are hand hygiene stations in all clinical areas				
14	There is hand hygiene poster(s) at the available Hand Hygiene station				
15	Hand hygiene observational audit conducted at least monthly for the past 3 months (Check the completed observation form and/or report)				
16	Soap or hand-rub alcohol is available at all Hand Hygiene stations				
17	There is water in the handwashing station during the visit				
18	There has not been an interruption to water supply for 1 day or more in the last 1 month in the facility				
**SCORE FOR Qs 13-18**		/6	5-6	4-3	<3
**RAG STATUS**					

IV. PERSONAL PROTECTIVE EQUIPMENT (PPE) & SUPPLIES

Instruction: *For supplies to be accessible at all times, stores are not locked or there is a functional system for gaining access when store keeper not there. If neither condition is met, score No (0).*

#	Standards	Score<br>No=0<br>Yes=1			
19.	Minimum stock of IPC supplies available at the time of visit *(See attachment for the minimum list of IPC supplies for certain period)*				
20.	All of the following PPE supplies are readily available for use<br>• *Elbow gloves*<br>• *Examination gloves*<br>• *Face shields*<br>• *Plastic apron*<br>• *Face mask*<br>• *Gowns*				
21.	Report, request and issue voucher (RRIV) completed and archived chronologically				
22.	There is a stock ordering book in use at the facility				
23.	PPE supplies are stored off the floor and in a dry place				
	**SCORE FOR Qs 19-23**	/5	4 -5	3	<2
	**RAG STATUS**				

V. SHARPS SAFETY

Instruction: *Sharps bins should be available in the clinic area, each treatment area, ward, single room or there should be a clear system of taking a sharps bin to the location of clinical activity when sharps are to be used. If not score No (0).*

#	Standards	Score<br>No=0<br>Yes=1			
24.	Syringes & Needles are discarded in sharp box after single use				
25.	There is sharp injury report for PEP management				
26.	Sharps containers are available in every clinical area				
27.	Sharp posters are clearly displayed where sharps are used/prepared				
28.	There are no sharps on the floor (i.e. needles, scalpels, ampoules, etc)				
29.	The sharps containers are not filled above the fill mark (not above 3/4 full)				
	**SCORE FOR Qs 24-29**	/6	5-6	3-4	<3
	**RAG STATUS**				

VI. DECONTAMINATION OF MEDICAL EQUIPMENT

Instruction: *Equipment waiting to be decontaminated should be clearly separated from equipment that has been cleaned / disinfected/ sterilised so there is no risk of cross contamination (one in use, one ready and the other one in sterilization process).*
A sterilisation unit should have an autoclave, medical equipment should be wrapped before being put in for sterilisation there should be some means of identifying sterilised equipment e.g. Bowie Dick tape that shows black stripes and sterile instruments should be stored in a clean dry area.

#	Standards	Score<br>No=0<br>Yes=1			
30.	There is a policy on cleaning, disinfection and/or sterilization of surgical instruments and other medical devices (Check for written policy)				
31.	There is a designated area / room for cleaning and disinfection of medical devices at the decontamination area				
32.	There is a separate designated area for inspection, assembly and packaging of medical devices at the decontamination area				
33.	There is a hand washing station in decontamination area				
34.	Staff in-charge of cleaning and disinfection of medical devices wears appropriate PPE				
35.	Staff handle dirty instruments with rubber gloves, plastic aprons, masks and gowns				
36.	There is at least one sterile delivery set at time of visit				
37.	There is a functional autoclave in use to sterilize critical medical devices e.g. forceps, scissors				
38.	The sterile packs are stored in a well-ventilated room or cabinet				
	**SCORE FOR Qs 30-38**	/9	8-9	5-7	<5
	**RAG STATUS**				

VII. DECONTAMINATION OF LINEN

#	Standards	Score<br>No=0<br>Yes=1	Comments		
39.	There is a designated laundry area				
40.	There is SOP for handling linen (*Check for physical copy of SOP)*				
41.	Soiled or dirty linens bagged at site of collection and is transported to a specific area in a closed ‘soiled linen’ bag/ container				
42.	Staff handle dirty linen are only by wearing Personal Protective Equipment (PPE)				
43.	Clean linen stored in an area separate from the storage of any dirty linens				
44.	Clean linen (including pillows) not stored on the floor, chairs, or counter tops				
**SCORE FOR Qs 39-44**		/6	5-6	3-4	<3
**RAG STATUS**					

VIII. DECONTAMINATION OF THE ENVIRONMENT

#	Standards	Score<br>No=0<br>Yes=1	Comments		
45.	The environment is visibly clean (Both inside and outside the facility; no litter scattered on floors or grounds, no spills seen on the floors)				
46.	There is a written schedule for decontamination of the facility at least twice a week (*Check for presence of written schedule and verify that up to date)*				
47.	Staff have knowledge of recommended environment decontamination procedures *(Ask staff to describe or demonstrate decontamination procedures and handling of contaminated materials)*				
48.	Staff have knowledge on making chlorine solutions for disinfection *(Ask staff to describe or demonstrate making the different concentrations of chlorine)*				
49.	Mattresses have intact waterproof covers or used with a separate mackintosh. *(Randomly select two wards (hospitals), if there is at least a single bed with no intact cover then score = 0. If mattress cover torn but staff use separate mackintosh to protect the cover then score = 1)*				
	**SCORE FOR Qs 45-49**	/5	4-5	3	<3
	**RAG STATUS**				

IX. WASTE MANAGEMENT

50.	The waste management policy (SOP) is available (Check for physical copy of SOP)				
51.	Waste is segregated at the point of care (point of generation)				
52.	Waste is disposed of according to appropriate colour coding				
53.	There are no overfull waste bins in the wards				
54.	There is appropriate bin liner in each bin (according to the colour coding policy)				
55.	Waste segregation posters are clearly displayed above all waste bins (The content list for waste segregation is posted on the wall)				
56.	All infectious or sharps waste is destroyed /collected from the facilities the same day				
57.	Verify existence of safe burning pit at the facility (fenced, brick enclosure at the ground, waste residues/ashes buried)				
58.	There is a functional placenta pit				
	**SCORE FOR Qs 50-58**	/9	8-9	5-7	<5
	**RAG STATUS**				

X. SANITATION

59.	All latrines are functional and clean with constant source of water				
60.	There are latrines clearly designated for each of the following groups: staff, and others (patients & visitors)				
61.	Separate toilets for women and men are available				
62.	There is at least one toilet accessible to people with reduced mobility				
63.	Signed record of cleaning available (*Check for physical copy of cleaning records)*				
64.	There is functional system for grey water drainage and soak away pit				
**SCORE FOR Qs 59-64**		/6	5-6	3-4	<3
**RAG STATUS**					

SUMMARY OF SCORES

#	Section Name	Total Score	Comments		
I.	SCREENING AND ISOLATION	/8			
II.	IPC/WASH INFORMATION	/4			
III.	HAND HYGIENE	/6			
IV.	PPE AND SUPPLIES	/5			
V.	SHARPS SAFETY	/6			
VI.	DECONTAMINATION OF MEDICAL EQUIPMENT	/9			
VII.	DECONTAMINATION OF LINEN	/6			
VIII.	DECONTAMINATION OF THE ENVIRONMENT	/5			
IX.	WASTE MANAGEMENT	/9			
X.	SANITATION	/6			
**SCORE FOR Qs 1-68 for PHUs**		/64	>85%	70-85%	<70%
**RAG STATUS for PHUs**			55-64	45-54	<45

Name of person interviewed	Contact Number	E-mail Address

Name of person interviewers, Organization	Contact Number	E-mail Address

Note

Purpose of the IPC/WASH Assessment tool: The purpose will be to examine the actual IPC/WASH practices at the facilities based on the written IPC policies and standards and improve health care service. Finally, it will also be used for risk assessment, root cause analysis and strategic planning.

How to use the audit tool: This tool is to be used by infection control practitioners and unit managers as well (or anyone trained on how to conduct IPC assessment).

Scoring

All criteria should be scored either ‘Yes’ for achieved (score ‘1’), ‘No’ for not achieved (score ‘2’). Scoring should be carried out as follows:-

Add the total number of ‘Yes’ answers and divide by the total number of questions answered (including all ‘Yes’ and ‘No’ answers) multiply by 100 to get the percentage.

Formula: $\frac{\text{Total number of yes answers}}{\text{Total number of yes and no responses}} \times 100 = \%$

Level of Compliance

Percentage scores can be allocated a level of compliance using the compliance categories below.

- Compliant: 85% or above (GREEN)
- Partial compliance: 70 to 84% (AMBER)
- Minimal compliance: below 70% (RED)

Each section within the audit tool will receive an overall score. This will identify any specific areas of partial or minimal compliance and will assist in the identification of areas were improvement is most required to ensure that the appropriate action is taken.

Auditing

The auditors obtain information from observations in functional areas including, direct questioning of staff (Nursing, Doctors, support staff and others), observation of IPC practices, inspection of environment cleanliness and review of relevant documentation. If any serious concerns are identified during the assessment, it should be brought to the attention of the person in-charge before the assessors leave the premises, and then reported to Infection Control Focal Person.

Feedback

Verbal feedback of key findings should be given to the person in-charge of the area prior to leaving or as soon as possible. A written copy of the findings and actions required should be made available to all relevant personnel within locally agreed timeline. A re-audit of a functional area may be undertaken if there are concerns or a minimal compliance rating is observed to ensure action has been taken.

Screening of inpatients should be performed twice daily and recorded for sign of any potential infectious disease.

Screening of staff should be undertaken at the start of each shift and there should be a system in place for following up staff who become ill to ensure they do not have an infectious disease or to ensure appropriate management if they do.

Table S2: National MOHS IPC/WASH standard health facility assessment checklist for Hospitals

GOVERNMENT OF SIERRA LEONE
MINISTRY OF HEALTH AND SANITATION
NATIONAL IPC/WASH HOSPITAL ASSESSMENT CHECKLIST

**Date of Assessment**	____/___/_______	**District**		**Chiefdom**	
**Facility Name**		**City/Town/Village**			

**Name & Designation of Person conducting Assessment**	**Contact Number**	**E-mail Address**

Type of facility

Hospital

Type of ownership

☐ Government ☐ Private ☐ Faith Based ☐ NGOs

**Name of Facility In-charge/Delegate**	**Contact Number**	**E-mail Address**

I. SCREENING AND ISOLATION

Instructions:

There should be a screening structure at the entrance to the facility or very close to it – if it is not immediately beside the entrance then score No (0)
Patients should only be able to access the facility by one entrance near or through the screening station – if there is more than one entrance and patients can enter without being screened then score No (0). There should be a hand hygiene station with either soap and water or hand sanitizer at all entrances – if either no soap or no water score as No (0)

**#**	**Standards**	**Score Yes=1 No=0**	**Comments**		
1	There is at least one screening station at the facility		I		
2.	All facility entrances have a screening station		I		
3.	The screening station has a single entrance		I		
4.	There is a hand hygiene station at all facility entry point		C		
5.	There is soap and water/Alcohol Based Hand Rub (ABHR) at the hand hygiene station		C		
6.	There is register/form with list of screened individuals		C		
7.	There is a designated isolation area		I		
8.	The isolation area is located in a permanent structure		I		
	**SCORE FOR Qs 1-8:**Total score out of 8	/8	7-8	5-6	<4
	**RAG STATUS**				

II. IPC/WASH ORGANIZATION

Instructions: *IPC and/or Wash focal person - either appointed to hospital or for PHU they know who the IPC focal person for their PHU cluster is or the IPC focal person at the DHMT – if they do not know who the IPC focal person is at the PHU score No (0).*
IPC committees – for Hospitals there should be a hospital committee, for PHUs there should be an IPC committee at the DHMT and the in-charge should be aware of this – if not score No (0).
An internal assessment tool e.g the ward assessment tool is in use as a regular monitoring tool for IPC compliance at the wards.
IPC committee minutes should be available from the Hospital IPC focal person or the DHMT focal person

**#**	**Standards**	**Score No=0 Yes=1**	**Comment**		
9.	There is a designated IPC and/or WASH focal person appointed for the facility<br>- *IPC Focal Person* □<br>- *WASH Focal Person* □		A		
10.	The IPC and/or WASH focal person received training based on the national IPC/WASH guidelines<br>- *IPC only* □<br>- *WASH only* □<br>- *IPC &WASH* □		A		
11	An IPC action plan was developed based on previous assessment feedback and was approved by facility leadership		A		
12	The action plan has been implemented (See report for activity progress)		A		
13	There is an IPC/WASH committee for the hospital		A		
14	The IPC/WASH committee meet every month. *(Tick/circle YES only if meeting minutes of last three (3) months are available)*		A		
15	The internal monitoring tool was completed in the last month for all wards *(Score '1' only if completed internal monitoring tool is available and verified as completed in last 30 days)*		A		
16	There is a completed screening form for in-patients in the wards for the last one month		A		
**TOTAL SCORE for Qs 9-16**		/8	7-8	5-6	<4
**RAG STATUS**					

III. HAND HYGIENE

Instruction: *If Hand Hygiene stations are not available in all clinic area, ward areas, and outside single rooms (e.g. labour rooms) -- then score No (0); If only available in some areas then score No (0). Minimum standard is one HH station per 10 patient beds.*
Monitoring Hand Hygiene compliance should be a formal system where opportunities for hand hygiene based on the five moments are observed for a period of at least 30 minutes on a regular basis in all the wards. If it is ad hoc, then score No (0).
A reliable water supply is > three days' supply with system of top up if provided in tanks or a backup system if there is interruption to the main supply.

#	Standards	Score No=0 Yes=1	Comment		
17	There are hand hygiene stations in all clinical areas		C		
18	There is hand hygiene poster(s) at the available Hand Hygiene station		C		
19	Hand hygiene observational audit conducted at least monthly for the past 3 months (Check the completed observation form and/or report)		A		
20	Soap or hand-rub alcohol is available at all Hand Hygiene stations		C		
21	There is water in the handwashing station during the visit		C		
22	There has not been an interruption to water supply for 1 day or more in the last 1 month in the facility		C		
**SCORE FOR Qs 17-22**		/6	5-6	4-3	<3
**RAG STATUS**					

IV. PERSONAL PROTECTIVE EQUIPMENT (PPE) & SUPPLIES

Instruction: *For supplies to be accessible at all times, stores are not locked or there is a functional system for gaining access when store keeper not there. If neither condition is met, score No (0).*

**#**	**Standards**	**Score**<br>**No=0**<br>**Yes=1**			
23.	Minimum stock of IPC supplies available at the time of visit *(See attachment for the minimum list of IPC supplies for certain period)*		C		
24.	All of the following PPE supplies are readily available for use<br>• *Elbow gloves*<br>• *Examination gloves*<br>• *Face shields*<br>• *Plastic apron*<br>• *Face mask*<br>• *Gowns*		C		
25.	Report, request and issue voucher (RRIV) completed and archived chronologically		A		
26.	There is a stock ordering book in use at the facility		C		
27.	PPE supplies are stored off the floor and in a dry place		A		
**SCORE FOR Qs 23-27**		/5	4 -5	3	<2
**RAG STATUS**					

V. SHARPS SAFETY

Instruction: *Sharps bins should be available in the clinic area, each treatment area, ward, single room or there should be a clear system of taking a sharps bin to the location of clinical activity when sharps are to be used. If not score No (0).*

#	Standards	Score No=0 Yes=1			
28.	Syringes & Needles are discarded in sharp box after single use		A		
29.	There is sharp injury report for PEP management		A		
30.	Sharps containers are available in every clinical area		C		
31.	Sharp posters are clearly displayed where sharps are used/prepared		C		
32.	There are no sharps on the floor (i.e. needles, scalpels, ampoules, etc)		A		
33.	The sharps containers are not filled above the fill mark (not above 3/4 full)		A		
**SCORE FOR Qs 28-33**		/6	5-6	3-4	<3
**RAG STATUS**					

VI. DECONTAMINATION OF MEDICAL EQUIPMENT

Instruction: *Equipment waiting to be decontaminated should be clearly separated from equipment that has been cleaned / disinfected/ sterilised so there is no risk of cross contamination (one in use, one ready and the other one in sterilization process).*
A sterilisation unit should have an autoclave, medical equipment should be wrapped before being put in for sterilisation there should be some means of identifying sterilised equipment e.g. Bowie Dick tape that shows black stripes and sterile instruments should be stored in a clean dry area.

**#**	**Standards**	**Score No=0 Yes=1**			
34.	There is a policy on cleaning, disinfection and/or sterilization of surgical instruments and other medical devices (Check for written policy)		A		
35.	There is a designated area / room for cleaning and disinfection of medical devices at the decontamination area		I		
36.	There is a separate designated area for inspection, assembly and packaging of medical devices at the decontamination area		I		
37.	There is a hand washing station in decontamination area		I		
38.	Staff in-charge of cleaning and disinfection of medical devices wears appropriate PPE		A		
39.	Staff handle dirty instruments with rubber gloves, plastic aprons, masks and gowns		A		
40.	There is at least one sterile delivery set at time of visit		C		
41.	There is a functional autoclave in use to sterilize critical medical devices e.g. forceps, scissors		I		
42.	The sterile packs are stored in a well-ventilated room or cabinet		I		
**SCORE FOR Qs 34-42**		/9	8-9	5-7	<5
**RAG STATUS**					

VII. DECONTAMINATION OF LINEN

#	Standards	Score<br>No=0<br>Yes=1	Comments		
43.	There is a designated laundry area		I		
44.	There is SOP for handling linen (*Check for physical copy of SOP)*		A		
45.	Soiled or dirty linens bagged at site of collection and is transported to a specific area in a closed 'soiled linen' bag/ container		I		
46.	Staff handle dirty linen are only by wearing Personal Protective Equipment (PPE)		A		
47.	Clean linen stored in an area separate from the storage of any dirty linens		A		
48.	Clean linen (including pillows) not stored on the floor, chairs, or counter tops		A		
**SCORE FOR Qs 43-48**		/6	5-6	3-4	<3
**RAG STATUS**					

VIII. DECONTAMINATION OF THE ENVIRONMENT

#	Standards	Score<br>No=0<br>Yes=1	Comments		
49.	The environment is visibly clean (Both inside and outside the facility; no litter scattered on floors or grounds, no spills seen on the floors)		A		
50.	There is a written schedule for decontamination of the facility at least twice a week (*Check for presence of written schedule and verify that up to date)*		A		
51.	Staff have knowledge of recommended environment decontamination procedures *(Ask staff to describe or demonstrate decontamination procedures and handling of contaminated materials)*		A		
52.	Staff have knowledge on making chlorine solutions for disinfection *(Ask staff to describe or demonstrate making the different concentrations of chlorine)*		A		
53.	Mattresses have intact waterproof covers or used with a separate mackintosh. *(Randomly select two wards (hospitals), if there is at least a single bed with no intact cover then score = 0. If mattress cover torn but staff use separate mackintosh to protect the cover then score = 1)*		C		
	**SCORE FOR Qs 49-53**	/5	4-5	3	<3
	**RAG STATUS**				

IX. WASTE MANAGEMENT

54.	The waste management policy (SOP) is available (Check for physical copy of SOP)		A		
55.	Waste is segregated at the point of care (point of generation)		A		
56.	Waste is disposed of according to appropriate colour coding		A		
57.	There are no overfull waste bins in the wards		A		
58.	There is appropriate bin liner in each bin (according to the colour coding policy)		C		
59.	Waste segregation posters are clearly displayed above all waste bins (The content list for waste segregation is posted on the wall)		C		
60.	All infectious or sharps waste is destroyed /collected from the facilities the same day		A		
61.	Verify existence of safe burning pit at the facility (fenced, brick enclosure at the ground, waste residues/ashes buried)		I		
62.	There is a functional placenta pit		I		
**SCORE FOR Qs 54-62**		/9	8-9	5-7	<5
**RAG STATUS**					

X. SANITATION

63.	All latrines are functional and clean with constant source of water		I		
64.	There are latrines clearly designated for each of the following groups: staff, and others (patients & visitors)		I		
65.	Separate toilets for women and men are available		I		
66.	There is at least one toilet accessible to people with reduced mobility		I		
67.	Signed record of cleaning available (*Check for physical copy of cleaning records)*		A		
68.	There is functional system for grey water drainage and soak away pit		I		
**SCORE FOR Qs 63-68**		/6	5-6	3-4	<3
**RAG STATUS**					

SUMMARY OF SCORES

#	Section Name	Total Score	Comments		
	SCREENING AND ISOLATION	/8			
	IPC/WASH INFORMATION	/8			
	HAND HYGIENE	/6			
	PPE AND SUPPLIES	/5			
	SHARPS SAFETY	/6			
	DECONTAMINATION OF MEDICAL EQUIPMENT	/9			
	DECONTAMINATION OF LINEN	/6			
	DECONTAMINATION OF THE ENVIRONMENT	/5			
	WASTE MANAGEMENT	/9			
	SANITATION	/6			
**SCORE FOR Qs 1-68 for the Hospitals**		/68	>85%	70-85%	<70%
**RAG STATUS for the hospitals**			58-68	48-57	<47

**Name of person interviewed**	**Contact Number**	**E-mail Address**

**Name of person interviewers, Organization**	**Contact Number**	**E-mail Address**

Note

Purpose of the IPC/WASH Assessment tool: The purpose will be to examine the actual IPC/WASH practices at the facilities based on the written IPC policies and standards and improve health care service. Finally, it will also be used for risk assessment, root cause analysis and strategic planning.

How to use the audit tool: This tool is to be used by infection control practitioners and unit managers as well (or anyone trained on how to conduct IPC assessment).

Scoring

All criteria should be scored either 'Yes' for achieved (score '1'), 'No' for not achieved (score '2'). Scoring should be carried out as follows:-

Add the total number of 'Yes' answers and divide by the total number of questions answered (including all 'Yes' and 'No' answers) multiply by 100 to get the percentage.

Formula: $\frac{\text{Total number of yes answers}}{\text{Total number of yes and no responses}} \times 100 = \%$

Level of Compliance

Percentage scores can be allocated a level of compliance using the compliance categories below.

- Compliant: 85% or above (GREEN)
- Partial compliance: 70 to 84% (AMBER)
- Minimal compliance: below 70% (RED)

Each section within the audit tool will receive an overall score. This will identify any specific areas of partial or minimal compliance and will assist in the identification of areas were improvement is most required to ensure that the appropriate action is taken.

Auditing

The auditors obtain information from observations in functional areas including, direct questioning of staff (Nursing, Doctors, support staff and others), observation of IPC practices, inspection of environment cleanliness and review of relevant documentation. If any serious concerns are identified during the assessment, it should be brought to the attention of the person in-charge before the assessors leave the premises, and then reported to Infection Control Focal Person.

Feedback

Verbal feedback of key findings should be given to the person in-charge of the area prior to leaving or as soon as possible. A written copy of the findings and actions required should be made available to all relevant personnel within locally agreed timeline. A re-audit of a functional area may be undertaken if there are concerns or a minimal compliance rating is observed to ensure action has been taken.

Screening of inpatients should be performed twice daily and recorded for sign of any potential infectious disease.

Screening of staff should be undertaken at the start of each shift and there should be a system in place for following up staff who become ill to ensure they do not have an infectious disease or to ensure appropriate management if they do
